# A zebrafish-centric approach to antiepileptic drug development

**DOI:** 10.1242/dmm.049080

**Published:** 2021-07-07

**Authors:** Scott C. Baraban

**Affiliations:** Department of Neurological Surgery and Weill Institute for Neuroscience, University of California, San Francisco, CA 94143-0350, USA

**Keywords:** Epilepsy, Perspective, Zebrafish

## Abstract

*Danio rerio* (zebrafish) are a powerful experimental model for genetic and developmental studies. Adaptation of zebrafish to study seizures was initially established using the common convulsant agent pentylenetetrazole (PTZ). Larval PTZ-exposed zebrafish exhibit clear behavioral convulsions and abnormal electrographic activity, reminiscent of interictal and ictal epileptiform discharge. By using this model, our laboratory developed simple locomotion-based and electrophysiological assays to monitor and quantify seizures in larval zebrafish. Zebrafish also offer multiple advantages for rapid genetic manipulation and high-throughput phenotype-based drug screening. Combining these seizure assays with genetically modified zebrafish that represent Dravet syndrome, a rare genetic epilepsy, ultimately contributed to a phenotype-based screen of over 3500 drugs. Several drugs identified in these zebrafish screens are currently in clinical or compassionate-use trials. The emergence of this ‘aquarium-to-bedside’ approach suggests that broader efforts to adapt and improve upon this zebrafish-centric strategy can drive a variety of exciting new discoveries.

## Abstract (precis)

**Summary:** Screening programs using zebrafish could reshape traditional approaches to antiepileptic drug discovery. Here we describe steps to develop zebrafish models that represent human epilepsies, with an emphasis on a zebrafish model of Dravet syndrome.

## Introduction

Epilepsy is a common neurological disorder with a variety of underlying etiologies. In adults, acquired epilepsies – resulting from traumatic brain injury (Annegers and Coan, 2000), a history of febrile seizures (Offringa et al., 1992) or infection (van Baalen et al., 2017) – are most common. Although exceptions exist, many adult epilepsy patients achieve adequate seizure control with antiepileptic drugs (AEDs) (Camfield and Camfield, 2008), vagal nerve stimulation (Nemeroff et al., 2006) or surgical resection (Barba et al., 2021). The success of AEDs in this population is likely to stem from the availability of 28 Federal Drug Administration (FDA)-approved drugs discovered in preclinical, primarily acute, rodent seizure models (Bourgeois, 1994; White, 1997; Loscher, 2002). Indeed, it can be argued that rodent seizure models, unlike those for Alzheimer's disease, stroke or spinal cord injury, are reasonably good preclinical predictors of AED activity. Nonetheless, consternation persists within the epilepsy community as the overall percentage of intractable epilepsy patients has remained in the 20–30% range for decades. Upon further reflection, maybe this is not surprising as none of the available rodent models commonly used to identify AEDs had been explicitly designed to represent pediatric forms of epilepsy, which specifically include a large number of rare genetic epilepsies associated with *de novo* single-gene mutations (Epi, 2012; Epi et al., 2013). Thus, FDA-approved AEDs for this population remain a significant unmet need, requiring alternative preclinical animal models.

“Zebrafish disease models can be used to evaluate a rapidly growing number of candidate genes for neurological disorders, including rare genetic epilepsies apparent in children.”

### Aquarium-to-bedside in epilepsy

Approaching this problem from a different perspective, we focused our laboratory efforts on pediatric epilepsies. At that time (in the early 2000s), existing animal models for pediatric epilepsies were primarily limited to rodent models of cortical malformation; for example undercut (Hoffman et al., 1994), freeze-lesion (Jacobs et al., 1996) or *in utero* methylazoxymethanol exposure (Baraban et al., 2000). Relatively little was known about genetic causes for epilepsies, grouped as ‘idiopathic’, as this predated widespread identification of gene mutations in humans and accompanying mutagenesis studies in mice. As most AED discovery efforts rely heavily on seizure thresholds in acute or invoked rodent models (Alachkar et al., 2020; Loscher, 2017), we sought preclinical models that feature a more clinically relevant phenotype of spontaneous unprovoked seizures (epilepsy) and amenability to high-throughput drug screening. Larval zebrafish, a small vertebrate widely used for neurodevelopmental research, featuring genetic homology with humans as high as 82% (Howe et al., 2013), fit this overall goal. However, although the concept of using zebrafish appeared attractive, experimental techniques to study seizures – at behavioral or electrophysiological levels – were lacking. To address this problem, with assistance from experienced zebrafish collaborator Herwig Baier, we established simple locomotion-based and extracellular electrophysiological recording techniques to monitor seizure events elicited by application of a well-established convulsant drug, pentylenetetrazole (PTZ) (Baraban et al., 2005). Early PTZ studies described progressive seizure-like behavioral stages in larval zebrafish and provided a clear demonstration of abnormal ictal- and interictal-like electrographic activity, both of which are cardinal features in defining epileptic conditions. Published in 2005 and presented to the epilepsy research community at national meetings, initial responses from more-senior epilepsy investigators and clinicians were not entirely supportive. Undaunted, we continued to publish manuscripts describing molecular, behavioral, electrophysiological and pharmacological features of larval zebrafish seizures for another decade (Baraban et al., 2007; Chege et al., 2012; Hortopan and Baraban, 2011; Hortopan et al., 2010; Hunt et al., 2012; Ramirez et al., 2012). Eventually, much of our early work with acute larval PTZ seizures was replicated in laboratories worldwide (Afrikanova et al., 2013; Baxendale et al., 2012; Orellana-Paucar et al., 2013; Siebel et al., 2015; Turrini et al., 2017; Copmans et al., 2018; Fuller et al., 2018; Tanwar et al., 2019; Bandara et al., 2020; Hoffman et al., 2016; Berghmans et al., 2007; Zheng et al., 2018; Pisera-Fuster et al., 2017), providing independent validation of this experimental model. Now, with more than 325 PubMed entries for “zebrafish+seizure”, acute zebrafish seizure models are well-established to study networks and mechanisms that result in the generation or propagation of seizures. From a pharmacological perspective, zebrafish PTZ seizures are similar to those in rodent models (Loscher, 2011; Alachkar et al., 2020) and identify the same AED candidates. As the database of drugs screened in rodent PTZ models dates to the 1940s, opportunities for new discoveries, in any species, using this convulsant agent may be limited.

**Fig. 1.**
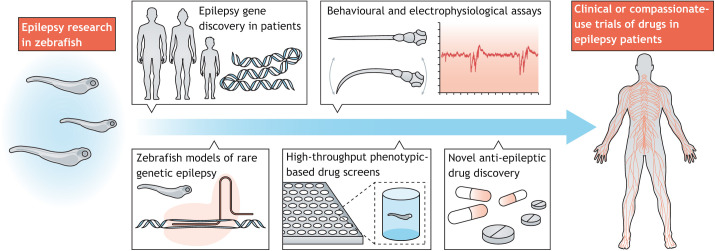
**Aquarium-to-bedside drug development for epilepsy.** Genes associated with rare genetic epilepsies, such as Dravet syndrome (DS), have been identified in patients. Genetically modified zebrafish models generated using ENU-mutagenesis or CRISPR-Cas9 technology can recapitulate these rare genetic epilepsies. Locomotion-based and local field potential (LFP) recording techniques adapted to larval zebrafish can then be used to study these rare genetic epilepsy models. Sensitive phenotype-based screening of hundreds to thousands of drugs can be achieved using a combination of these behavioral and electrophysiological assays in genetically modified zebrafish models, and use of a DS zebrafish model identified several drugs that progressed directly into clinical or compassionate-use trials.

Zebrafish disease models can be used to evaluate a rapidly growing number of candidate genes for neurological disorders, including rare genetic epilepsies apparent in children. These discoveries benefit from several advantages that are unique to this simple vertebrate system, including – but not limited to – a fully sequenced genome, rapid neurodevelopment and large clutches of transparent embryos that develop externally. Most relevant to neurological disorders, the main central nervous system subdivisions are similar to those in mammals, i.e. forebrain, midbrain, hindbrain and spinal cord, as are the common neurotransmitter systems, i.e. γ–aminobutyric acid (GABA), glutamate, dopamine, norepinephrine, serotonin and acetylcholine (Choi et al., 2021). A decades long history of generating mutants by using the chemical mutagenesis agent N-ethyl-N-nitrosourea (ENU) combined with advancements in transposons, transcription activator-like effector nuclease (TALEN) and Zinc-finger nuclease (ZFN) genome editing resulted in hundreds of zebrafish lines (Huang et al., 2012; Auer and Del Bene, 2014; Ata et al., 2016; Thyme et al., 2019). More recently, the CRISPR/Cas9 system was shown to be several-fold more efficient at generating germline mutations, resulting in several clinically relevant zebrafish lines that mimic known human epilepsy mutations (Grone et al., 2017; Pena et al., 2017; Hoffman et al., 2016). As CRISPR technology was unavailable until recently, our initial drug program utilized an ENU-generated mutant for the voltage-activated sodium channel SCN1A that is associated with Dravet syndrome (DS), one of the most-severe forms of genetic epilepsy. The *didy^s552^*/*scn1lab* larval zebrafish mutants mimic a haploinsufficient *SCN1A* loss-of-function mutation seen in most DS patients. Following identification by Herwig Baier's laboratory in a large-scale screen for saccade deficits (Schoonheim et al., 2010), we determined that *scn1lab* mutant larvae exhibit frequent and recurrent spontaneous seizures early in development, are not associated with genetic compensation by other *scn1* genes, die prematurely between 10 and 14 days post fertilization (Baraban et al., 2013), and experience sleep-wake cycle disturbances (Grone et al., 2017) as well as metabolic deficit (Banerji et al., 2021; Kumar et al., 2016). Seizures in *scn1lab* mutant larvae respond favorably to DS ‘standard of care’ AEDs valproate, benzodiazepines and stiripentol but are pharmaco-resistant to most other AEDs (Baraban et al., 2013; Griffin et al., 2018; Griffin et al., 2019; Banerji et al., 2021; Dinday and Baraban, 2015; Griffin et al., 2017; Hong et al., 2016). Many of these phenotypes, including spontaneous epileptic phenotypes at behavioral and electrophysiological levels, were subsequently replicated by other laboratories using *didy^s552^*/*scn1lab*, CRISPR-generated *scn1lab* or morpholino antisense *scn1lab* knockdown larval zebrafish (Sourbron et al., 2017; Sourbron et al., 2016; Sourbron et al., 2019; Eimon et al., 2018; Weuring et al., 2020). These unique features recapitulate key clinical phenotypes and allow for high-throughput drug screening in larval zebrafish.

“By using a two-stage locomotion and electrophysiology phenotype-based screening strategy, and highlighting the predictive value of our DS zebrafish mutant, we identified fenfluramine […], synthetic cannabinoids […], structural analogs of clemizole and – as repurposed drugs – trazodone and lorcaserin.”

With the establishment of this genetic zebrafish model, and in combination with optimized simple locomotion-based and electrophysiological recording assays, we initiated our first phenotype-based drug-screening program using a commercially available 312 compound drug library spiked with FDA-approved drugs (Baraban et al., 2013). As FDA-approved treatments for DS were unavailable when we started this research in 2012, we reasoned that a repurposed drug library could yield a lead candidate(s) permissive to rapid clinical translation. In the initial phenotype-based screen, now expanded to over 3500 drugs (Griffin et al., 2020, 2017, 2019; Dinday and Baraban, 2015; Banerji et al., 2021), we identified the first-generation antihistamine clemizole (EPX-100) to be a powerful inhibitor of spontaneous recurrent seizures. We also learned that, by itself, a simple locomotion-based assay yielded ‘false positive’ results of drugs that reduced seizure-like behavior but did not change electrographic events because they are sedatives or muscle-relaxants. It is worth a strong note of caution that many subsequent screening efforts that have adapted our larval zebrafish seizure models largely relied upon locomotion-based outcomes but lack any electrophysiological confirmation of anti-seizure activity, thereby adding putative ‘hit’ compounds to the literature that are likely to be false positives (Sharma et al., 2020; Shen et al., 2020; Gong et al., 2020; Baxendale et al., 2012; Jones et al., 2020; Thornton et al., 2020; Brillatz et al., 2020; Li et al., 2020; Decui et al., 2020; Plate et al., 2019; Brueggeman et al., 2019; Aourz et al., 2019; Bertoncello et al., 2018; Moradi-Afrapoli et al., 2017; Li et al., 2015; Siebel et al., 2015; Rahn et al., 2014; Buenafe et al., 2013). Specifically, acute larval PTZ-induced seizure assays coupled with electrophysiological confirmation can accurately predict AED efficacy (Baraban et al., 2005; Afrikanova et al., 2013; Berghmans et al., 2007), whereas behavior-only discoveries lacking this crucial electrophysiology assay are difficult to interpret. By using a two-stage locomotion and electrophysiology phenotype-based screening strategy, and highlighting the predictive value of our DS zebrafish mutant, we identified fenfluramine (now FDA-approved as Fintepla^®^) (Dinday and Baraban, 2015), synthetic cannabinoids (similar to the FDA-approved cannabadiol Epidiolex^®^) (Griffin et al., 2020), structural analogs of clemizole and – as repurposed drugs – trazodone (Desyrel^®^) and lorcaserin (Belviq^®^) (Griffin et al., 2017). Interestingly, and suggesting an additional advantage of a zebrafish model, we combined medicinal chemistry, receptor binding assays and *scn1lab* mutant assays to identify a potentially unifying mechanism of action of these drugs, which is linked to the modulation of serotonin receptors (Griffin et al., 2019). Although innovative from a preclinical model perspective and accomplished in less than a decade, the fact likely to have brought this zebrafish model to the attention and acceptance of the epilepsy community is that, during compassionate-use trials, lorcaserin and, to a lesser extent, trazodone have both shown efficacy against seizures in children suffering from DS (Griffin et al., 2017; Tolete et al., 2018; Brigo et al., 2018). Moreover, clemizole (EPX-100), which exhibited a broad safety profile in recent phase I clinical studies is now under investigation as an ‘add-on treatment’ in a pivotal phase II clinical trial (https://clinicaltrials.gov/ct2/show/NCT04462770).

## Conclusion

Our success with a larval zebrafish model for DS is but one small example of what may be possible. Although this system may not easily adapt to modeling forms of epilepsy that are acquired in adulthood (as many advantages inherent in using larvae are lost in adult zebrafish) or, indeed, all genetic forms of epilepsy (as zebrafish orthologs for every human gene may not be available), zebrafish models could offer similar routes to drug discovery for personalized medicine. In line with this, we recently used CRISPR/Cas9 genome editing to generate 37 stable mutant zebrafish lines that represent a wide spectrum gene mutations important regarding human epilepsy (https://zebrafishproject.ucsf.edu/) (Griffin et al., 2020). We envision these zebrafish models, which are based on loss-of-function mutations found in humans, as a starting point for future studies aiming to better understand how a single-gene mutation leads to altered network function and, ultimately, intractable forms of epilepsy. For example, mechanistic insights into seizure generation and/or propagation may be possible when using cutting-edge imaging techniques (Dana et al., 2016; Marvin et al., 2019; Shemesh et al., 2020; Liu et al., 2021) combined with opto- or chemogenetic manipulation of neuronal sub-populations. In addition to drug discoveries as described above, genetic zebrafish lines also offer opportunities to design novel phenotype-based screening strategies for preclinical development of targeted therapies based on antisense-oligonucleotide, CRISPR-Cas9 or adeno-associated viral vector gene manipulation technologies (Han et al., 2020; Colasante et al., 2020; Niibori et al., 2020). More broadly, as a challenge for the zebrafish community, our epilepsy-specific strategy might be adaptable to a wider variety of neurological disorders. In consideration of these challenges and our experience, we suggest that scientists seeking alternative approaches to rodent preclinical models should seriously consider zebrafish – as zebrafish offer many advantages for the potential identification of new treatments for genetic diseases.
